# The influence of potential stressors on oviposition site selection and subsequent growth, survival and emergence of the non‐biting midge (*Chironomus tepperi*)

**DOI:** 10.1002/ece3.5148

**Published:** 2019-04-10

**Authors:** Robin Hale, Valentina Colombo, Molly Hoak, Vin Pettigrove, Stephen E. Swearer

**Affiliations:** ^1^ School of BioSciences University of Melbourne Parkville Victoria Australia; ^2^ Centre for Aquatic Pollution Identification and Management Parkville Victoria Australia

**Keywords:** Chironomidae, ecological trap, fitness, natal habitat preference induction, oviposition, salinization

## Abstract

Theory predicts that animals should prefer habitats where their fitness is maximized but some mistakenly select habitats where their fitness is compromised, that is, ecological traps. Understanding why this happens requires knowledge of the habitat selection cues animals use, the habitats they prefer and why, and the fitness costs of habitat selection decisions. We conducted experiments with a freshwater insect, the non‐biting midge *Chironomus tepperi* to ask: (a) whether females respond to potential oviposition cues, (b) to explore whether oviposition is adaptive in relation to metal pollution and conductivity, and (c) whether individuals raised in poor quality sites are more likely to breed in similarly poor locations. We found the following: (a) females responded to some cues, especially conductivity and conspecifics, (b) females preferred sites with higher concentrations of bioavailable metals but suffered no consequences to egg/larval survival, (c) females showed some avoidance of high conductivities, but they still laid eggs resulting in reduced egg hatching, larval survival, and adult emergence, and (d) preferences were independent of natal environment. Our results show that *C. tepperi* is susceptible to ecological traps, depending on life stage and the relative differences in conductivities among potential oviposition sites. Our results highlight that (a) the fitness outcomes of habitat selection need to be assessed across the life cycle and (b) the relative differences in preference/suitability of habitats need to be considered in ecological trap research. This information can help determine why habitat preferences and their fitness consequences differ among species, which is critical for determining which species are susceptible to ecological traps.

## 1 INTRODUCTION

Habitat selection can have profound effects on population growth, influence species interactions, and determine assemblage composition (Morris, 2003). Many animals use indirect cues that indicate current and likely future conditions (e.g., food resources) to select habitats (Orians & Wittenberger, 1991), based on the presumption that these cues help them identify locations where their fitness (i.e., survival, reproduction) is maximized. However, when the environment changes rapidly (e.g., from human‐induced rapid environmental change: HIREC; Sih, Ferrari, & Harris, 2011; Sih, 2013), the links between habitat quality and preference can be decoupled. Some animals then become caught in ecological traps, when they mistakenly select habitats where their fitness is lower than if they had chosen another option (Robertson & Hutto, 2006). Ecological traps affect a variety of taxa (Hale & Swearer, 2016; Robertson, Rehage, & Sih, 2013). Perhaps the most compelling examples are aquatic insects that are attracted to artificial surfaces (e.g., roads, buildings) reflecting polarized light more strongly than streams (e.g., Horvath, Malik, Kriska, & Wildermuth, 2007), where they lay their eggs which subsequently die.

Research has focused more on documenting ecological traps than on understanding the sensory or cognitive mechanisms underpinning them (Robertson & Chalfoun, 2016). Information about habitat selection behavior will help understand why some animals respond maladaptively to HIREC (Sih, Trimmer, & Ehlman, 2016). Species with poorer knowledge of the environment (Battin, 2004), such as being less responsive to environmental cues, or less choosy between habitats (Hale, Coleman, Sievers, Brown, & Swearer, 2018), are more likely to behave suboptimally. Conversely, animals with more complicated behaviors may be better informed, such as using multiple cues (Huijbers et al., 2012) potentially with different sensory modalities (Munoz & Blumstein, 2012) and thus are less susceptible. Diversity in cue use can be related to the grain in evolved habitat selection behavior, with “coarse grained” species more selective about habitats than “fine‐grained” species (Rosenzweig, 1981), and thus more likely to adaptively select habitats. Having flexible habitat selection behaviors may also be beneficial, such as tungara frogs (*Physalaemus pustulosus*) that can adjust their oviposition strategies for different ecological contexts (Marsh & Borrell, 2001).

Natal experience can have strong influences not only on habitat selection behavior but also how ecological traps affect animals. Some species prefer similar sites to where they are born (natal habitat preference induction, or NHPI: Davis & Stamps, 2004), which can have benefits such as reducing searching time (Davis & Stamps, 2004). NHPI can lessen the effects of ecological traps (Kokko & Sutherland, 2001), as reproductive output is increased in higher quality habitats, which more animals then select later in life. NHPI, however, is not always adaptive, for example, birds that breed in natal‐like habitats can have reduced reproductive success or output (Fletcher et al., 2015; Piper, Palmer, Banfield, & Meyer, 2013), if natal‐like habitats are of poor quality (e.g., low food resources). Animals born in suboptimal natal‐like habitats may select these later in life compounding the effects of ecological traps.

We conducted experiments to examine habitat selection (oviposition) of the non‐biting midge (*Chironomus tepperi*) and its consequences for their fitness. *C. tepperi* is a widespread Australian chironomid, with aquatic larval and flying adult life‐cycle stages. Adults rapidly colonize newly flooded habitats (Stevens, Warren, & Braysher, 2003), with females depositing egg ropes contained in jelly on the surface of the water (Martin & Porter, 1978). *C. tepperi* is an ideal model species: it is widely distributed and lives in ephemeral habitats where environmental conditions are likely to fluctuate and females generally only mate once (Martin & Porter, 1978), so there is likely to be strong selection on oviposition behavior.

Our three aims were to:

1. Test whether *C. tepperi* respond to potential oviposition cues;
2. Explore whether female *C. tepperi* make adaptive habitat selection decisions; and
3. Test whether natal habitat preference induction can exacerbate the effects of ecological traps, by individuals that are born into poor fitness locations then choosing similar ones to breed in.

## 2 MATERIALS AND METHODS

### 2.1 Overall strategy

Previous work has shown that female *C. tepperi* respond to nitrogenous compounds and different bioextracts during oviposition (Stevens et al., 2003), and other chironomids respond to polarized light (Lerner et al., 2008). To address Aim 1, we first tested whether they respond to humic acid (HA), which could be used by females to avoid habitats that have not recently flooded, given it is produced as organic matter decays and is higher in lentic habitats (Steinberg, 2003). We then tested whether they respond to visual and chemical cues from conspecific eggs, and whether these responses are context (i.e., density)‐dependent. Cues from conspecifics could indicate a habitat is suitable or be used by females to avoid areas where intraspecific interactions between larvae are intensified (Raitanen, Forsman, Kivelä, Mäenpää, & Välimäki, 2014). Thus females may benefit if they are able to respond to cues from conspecifics in different ways depending on the fitness costs of these decisions, for example, by being attracted to conspecifics at low densities, but then avoiding them as density increases. *C. tepperi* would be more likely to exhibit adaptive habitat selection behavior if they respond to multiple cues, especially using multiple sensory modalities, and this response is context‐dependent.

Theory predicts that ecological traps are most likely in scenarios when the environment is changing rapidly and animals are exposed to evolutionary novel situations (Sih, 2013). With this in mind and to address Aim 2, we exposed *C. tepperi* to water from four urban stormwater treatment wetlands, two of which are ecological traps for frogs (Sievers, Parris, Swearer, & Hale, 2018), and tested whether fitness and preference vary among habitats. Stormwater wetlands are being constructed around many cities to treat polluted stormwater runoff (Malaviya & Singh, 2012), and these wetlands may have high loads of novel pollutants (i.e., synthetic biocides) but also characteristics of “high quality” habitats (e.g., native vegetation), creating a mismatch between habitat suitability and preference (e.g., Tilton, 1995; Hale, Coleman, Pettigrove, & Swearer, 2015). Stormwater pollutants found at our wetlands can hinder the ability of tadpoles to respond to habitat selection cues (Sievers, Hale, Swearer, & Parris, 2018). While chironomids are commonly used in standard pollution toxicity testing (e.g., OECD, 2010), they may suffer similar effects causing ecological traps. If some wetlands are traps, females would prefer or equally prefer more polluted sites where egg hatching success is reduced (i.e., "severe" or "equal preference" traps: Robertson & Hutto, 2006).

Following this initial work, we conducted a more consolidated series of experiments focusing on the effects of conductivity on *C. tepperi*. We tested the oviposition preferences of *C. tepperi* and their fitness when exposed to a gradient of environmentally relevant salinities (i.e., both Aims 1 and 2). Humans are introducing novel stressors to ecosystems, but also intensifying natural stressors that animals may have evolved some resistance to; salt is a natural component of freshwater ecosystems but increased salinization from anthropogenic sources is a global environmental issue (Cañedo‐Argüelles et al., 2013; Kefford et al., 2016). Hereafter, we refer to salinity as conductivity, which is its most common measure (Kefford, Nugegoda, Zalizniak, Fields, & Hassell, 2007). *C. tepperi* is distributed across Australia, has been recorded at ~70,000 μS/cm (Kefford, Dalton, Palmer, & Nugegoda, 2004), and is found commonly around Melbourne, where wetlands can be ~20,000 μS/cm (Carew, Pettigrove, Cox, & Hoffmann, 2007). Egg (Kefford et al., 2004) and larval (Hale, Marshall, Jeppe, & Pettigrove, 2014) survival can be negatively correlated with conductivity so there may be costs if females do not avoid high conductivities. Other insects can respond to conductivity when laying eggs (e.g., mosquitos prefer freshwater; Osborn, Diaz, Gomez, Moreno, & Hernandez, 2006) and have sensitive odor and taste receptors (Hallem, Dahanukar, & Carlson, 2006). *C. tepperi* thus may be able to detect conductivity, since they lay egg ropes at or near the water surface (Martin & Porter, 1978).

We used conductivity as the focus in a final set of experiments to address Aim 3. We raised midges at one conductivity where fitness was high, and another where fitness was lower, and then assessed their oviposition preferences. While NHPI is often tested in relation to neutral signals separate from the quality of the environment, we were specifically interested in examining the potential for maladaptive habitat selection when the cue has a direct fitness consequence. If midges are exhibiting NHPI, we predicted that females would lay their eggs in conductivities similar to where they were born, regardless of whether these are the most suitable habitats.

### 2.2 Test organisms

*Chironomus tepperi* is a short‐lived (ca. 21 days at 20°C) holometabolous insect. Females mate once and when not provided with food usually oviposit 1–2 egg masses with comparable hatching success (Martin & Porter, 1978). Females deposit eggs contained in jelly egg ropes in water 1–2 days after emergence, which typically hatch within 48 hr in laboratory conditions. Midges were collected from two field populations (one at the Yanco Agricultural Institute and the other at Rockbank, 37°44'59.9"S 144°42'07.8"E), both of which had bred for >5 generations in cultures maintained at the University of Melbourne, and thus are considered to be a homogenous laboratory culture. To address our three aims, we used laboratory choice experiments to examine the oviposition preferences of females and evaluated the fitness consequences of these decisions.

### 2.3 Laboratory choice experiments

We conducted choice experiments following a similar methodology to Stevens et al. (2003), with the following steps: (a) experimental treatments were prepared (water containing different visual and chemical stimuli, or water from different sites, depending on the experiment as outlined below), (b) petri dishes (120 mm diameter) were added to choice arenas (plexiglass cages 30 × 30 × 30 cm), with one petri dish of each treatment placed in the arena (in the corners for experiments with two/four treatments, corners and middle for experiments with five treatments, with position randomized), (c) 25–30 adults (roughly balanced sex ratio) were randomly collected from the main culture and placed into each cage within 10 min of treatments being added. Previous studies have anaesthetized adults to ensure exact numbers and sex ratios of individuals are tested (Stevens et al., 2003) but we were concerned that this may have affected adult behavior, (d) after 48 hr, the number of egg ropes laid in each petri dish (i.e., treatment) within each cage was recorded, and (e) adults were removed from the choice arena, which was then cleaned with ethanol between trials. Chironomid oviposition preference is studied in groups (e.g., Stevens et al., 2003; Lerner et al., 2011) as it is necessary for adults to mate before females lay their eggs. Adults die within 2–3 days of spawning so were only used once in experiments. To remove the potential for cage effects, we ran experiments over several days (Table 1), with multiple replicate trials beginning on each day (a replicate trial refers to one choice arena containing one petri dish per treatment). Tests were performed at 22°C (±1) under a 16:8 hr light:dark photoperiod, with a twilight time of 30 min before and after the light period (when *Chironomus* spp. are most active).

**Table 1:** (a) Details of choice experiments and (b) Egg hatching experiments

(a) Choice experiments			
Experiment	Treatments	Start date	Number of replicates
Aim 1	Humic acid	1/11/17	8
		6/11/17	8
		8/11/17	8
	Conspecific	5/10/16	10
		12/10/16	7
		04/11/16	5
Aim 2	Wetland choice	22/01/2018	8
		24/01/2018	8
		26/01/2018	7
Aim 2	Conductivity	25/10/16	9
		27/10/16	6
		28/08/17	8
		30/08/17	8
		05/09/17	8
Aim 3	NHPI	20/11/16	23 raised in 200 μs/cm
			19 raised from 5,000 μs/cm

(b) Egg hatching			
Experiment	Treatment		Number of replicates
Aim 2			
Eggs collected 24/01, 28/01	Replicates of water from all four wetlands		10 of each wetland
Aim 3			
	Conductivity		
2016	200 μs/cm		21
Eggs collected 26/10 and 27/10	1,000 μs/cm		16
	5,000 μs/cm		15
	10,000 μs/cm		15
2017	4,000 μs/cm		8
Eggs collected 27/10 and 30/08	6,000 μs/cm		8
	8,000 μs/cm		11
	10,000 μs/cm		12

Note

In the choice experiments, one replicate is an experimental tank containing one petri dish containing the different treatment options. For example, for the humic acid experiment, each tank contained two petri dishes, one with synthetic water with humic acid added and one just with synthetic water. In the egg hatching experiment, one replicate is a well plate containing one egg rope.

To address Aim 1, we ran two experiments to examine adult responses to cues from HA and from conspecifics. In the first, we gave adults a choice between two treatments: synthetic water, and synthetic water containing 200 mg/L HA. The synthetic water contained 18 g/L MgC_l2_·6H_2_O, 10 g/L CaCl_2_·2H_2_O, 10 g/L MgSO_4_, 2 g/L KH_2_PO_4_,10 g/L NaHCO_3_, 50 g/L NaCl and 0.2 mg/L 13% w/w Fe‐EDTA (ethylenediaminetetraacetic acid iron complex, Manutec) solution giving a final water hardness of 24 (±3.7) mg/L CaCO_3_, pH 6.4, and conductivity of 215–240 mS/cm and is used as stock solutions by the Centre for Aquatic Pollution Identification and Management (Colombo, Pettigrove, Hoffmann, & Golding, 2016). We are unaware of any studies that have examined the potential for HA to affect oviposition but this concentration has been shown to affect fish responses to olfactory cues (Fisher, Wong, & Rosenthal, 2006), and is within the range observed in freshwater habitats (Steinberg, 2003). We ran 24 replicate trials in November 2017 (Table 1). For the conspecific experiment, adults were given a choice between five treatments: synthetic water alone, synthetic water with one egg rope, synthetic water with five egg ropes, egg‐scented water with no egg ropes, and egg‐scented water with five egg ropes. To collect egg ropes and egg‐scented water, we established a cage with ~2 L of synthetic water and ~50 adults of roughly equal sex ratios. We collected egg ropes laid overnight in this cage, and water that had contained egg ropes using a pipette. Petri dishes were filled with egg‐scented synthetic water for these treatments. Other treatments were filled with synthetic water. We ran 22 replicates in October/November 2017 (Table 1).

For Aim 2, we collected water from four stormwater treatment wetlands located in the south‐eastern suburbs of the city of Melbourne, south‐eastern Australia. Two of these sites, Chandler Road (38°00’00.56”S, 145°10’46.19”E) and Cheltenham Road (37°59’27.68”S, 145°09’12.58”E), had high concentrations of bioavailable metals in sediments (e.g., zinc 2,390–3,790 mg/kg), and the other two Lynbrook Estate (38°03’20.84”S, 145°15’03.89”E) and Woodlands Lake (38°00’11.49”S, 145°07’18.82”E) wetlands had relatively lower concentrations (e.g., zinc 209–606 mg/kg). For detailed summaries of metal and pesticide concentrations in sediment at these sites, and other environmental variables, see Sievers, Parris, Swearer, et al. (2018). We collected water in 20 L carboys, and in each trial gave females a choice between four petri dishes each filled with water from one of the study sites. We ran 23 replicate trials in January 2018 (Table 1).

For the conductivity experiments (Aim 2), each cage contained four petri dishes which were randomly assigned conductivity treatments, created by adding NaCl to synthetic water. We gave adults a choice between waters of 200, 1,000, 5,000, and 10,000 μS/cm in 2016 and 4,000, 6,000, 8,000, and 10,000 μS/cm in 2017. We selected these concentrations based on work demonstrating that conductivities >3,000 μS/cm reduced larval survival, and >1,000 μS/cm slowed emergence (Hale et al., 2014). We used NaCl to manipulate conductivity while keeping all other constituents of the water constant. We ran 15 replicate trials in October 2016 and 24 in August/September 2017 (Table 1).

### 2.4 Fitness costs of habitat selection decisions

We examined the hatching rates of egg ropes as an endpoint to estimate fitness in Aims 2 and 3. Approximately 50 adults (with roughly balanced sex ratios) were added to Plexiglass cages which were filled to ~10 cm water depth with water from the four different source wetlands (Aim 2), or water with different conductivities (Aims 2 and 3). After 24 hr, egg ropes were collected and distributed randomly into well plates, so each well plate was filled with one egg rope and water from the treatment cage the egg was laid in (i.e., female oviposition preference was preserved), hereafter a “replicate.” We scored if egg ropes had hatched after 48 hr (usual timing of hatch: Kefford et al., 2004), based on viewing under an Olympus SZX7 microscope. To remove the potential for a cage artifact, egg ropes in all experiments came from multiple cages for each treatment, either established on the same day, or from several dates (see Table 1).

For the conductivity experiments in Aim 2, we also conducted larval and emergence bioassays in 2017 at 21 (±1)°C with basic water quality parameters (pH, dissolved oxygen, conductivity) monitored throughout. Methods followed those used by the Centre for Aquatic Pollution Identification and Management, based on standard approaches developed for sediment toxicity tests by OECD (2010). For growth and survival assays, 5‐day‐old larvae (raised in synthetic water, i.e., the stock solutions used above) added to eight replicate beakers (10 larvae per beaker) containing 400 ml of synthetic water. All surviving larvae were removed after 5 days, counted, and weighed (after being dried in a 60°C oven for 24 hr). Larvae were exposed to four conductivity treatments, matching those used in the 2017 choice experiments (i.e., 4,000, 6,000, 8,000, and 10,000 μS/cm) and prepared as above. For emergence assays, four replicate beakers of the 2,000, 4,000, and 8,000 μS/cm were prepared and incubated for up to 18 days, with the number of emerging adult *C. tepperi* counted daily.

### 2.5 Test of NHPI

To test for NHPI (Aim 3), we raised egg ropes in two conductivities (200 and 5,000 μS/cm). We selected 200 μS/cm as cultures were maintained at this conductivity, and 5,000 μS/cm as egg ropes hatched but later life‐cycle stages suffered some reduced fitness when exposed to 6,000 μS/cm (see Section 3). Adults were collected from the culture (i.e., had not previously been used in experiments), released into four breeding cages, with two replicate cages of each of the two conductivity treatments, and left to mate overnight. The following day, six egg ropes were harvested and placed into a breeding cage matching that respective treatment, with two breeding cages established for each treatment. Raising conditions followed earlier studies (Colombo, Pettigrove, Golding, & Hoffmann, 2014; Townsend, Pettigrove, & Hoffmann, 2012), and when adults emerged, we recorded their oviposition preference when offered waters with varying conductivities (200, 1,000, 5,000, and 10,000 μS/cm) using the choice experiment protocols outlined above.

### 2.6 Statistical analysis

For logistical reasons (e.g., access to laboratory space, choice cages, breeding adults), choice experiments were repeated multiple times (Table 1). We initially ran mixed effects models for each choice experiment testing if the predictor variable (mean numbers of egg ropes laid per treatment, which was log‐transformed to improve normality) varied between treatments. We included Experimental Run (i.e., first, second, third) and Breeding cage as two random factors, with the total numbers of eggs laid in each cage used to weight the response. All statistical tests were performed in R (R Development Core Team, 2017), with these models fitted using the lmer function from the lme4 package, with weights implemented using the “weights” argument. In all choice experiments, the two random factors (Experimental Run and Breeding cage) explained a very low amount of the variance (1%–7%). We therefore tested for treatment effects using generalized linear models (using the glm function), with Treatment as a categorical predictor, and the response variable weighted as per the mixed effects model. In all cases, there was no statistical difference in the fit of the linear mixed effects and generalized linear models (*p* > 0.05 in all cases), which was tested using the ANOVA function. For the generalized linear models, we also used the ANOVA function to conduct an *F*‐test and extract a *p*‐value to test for the overall statistical significance of the treatment effect, and conducted post hoc Tukey's tests to explore differences between treatments using the glht function from the multcomp library. Given that midges were exposed to different conductivity treatments in 2016 and 2017, we ran separate models for the 2 years rather than running a mixed effects model with year as a random effect. We did this as a more conservative approach to account for the possibility that the behavior of females is affected by the relative differences among the options they are assessing; a mixed effects model would not allow this to be evaluated. We analyzed the natal habitat preference induction experiment using a generalized linear model with Treatment and Natal Experience as two factors, after first running a mixed effect model that also included Breeding Cage as a random effect as above (this explained <1% of the variance).

We fitted a dose–response curve to relate egg rope hatching rates to conductivity (Aim 2), using a 3‐parameter log‐logistic function with parameters estimated by maximum likelihood (Ritz & Streibig, 2005). To compare if other larval and emergence endpoints differed across conductivity treatments, we used a one‐way ANOVA to describe overall effects and Tukey's HSD to examine which levels of each treatment were significantly different.

## 3 RESULTS

### 3.1 Aim 1: Do *C. tepperi* respond to potential oviposition cues?

**Figure 1:**
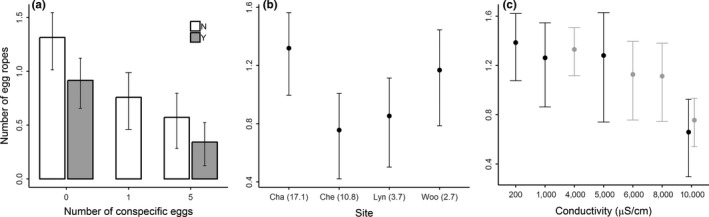
Oviposition of the non‐biting midge *Chironomus tepperi* in response to (a) eggs from conspecifics, (b) water collected from four different stormwater wetlands around the city of Melbourne, Australia, and (c) conductivity. In (a), the *x*‐axis illustrates the number of conspecific eggs in each treatment, and whether eggs were placed into egg‐scented water (Y = yes, gray bars) or synthetic water (N = no, white bars). In (b), Cha = Chandler, Che = Cheltenham, Lyn = Lynbrook, Woo = Woodlands. Chandler and Cheltenham are two more polluted sites that have been shown to be ecological traps for spotted marsh frog *Lynodynastes tasmaniensis* when compared to Lynbrook and Woodlands, with the number in parenthesis showing the heavy metal quotient at each site (Sievers, Parris, Swearer, et al., 2018), which is an integrative measure of likely pollutant levels. In (c), black and gray circles indicate data from 2016 and 2017, respectively. In all plots, the response variable is the mean number (±95% confidence interval) of egg ropes laid in each treatment across all replicates, which was log‐transformed

Females laid comparable numbers of egg ropes in synthetic water versus synthetic water with HA (generalized linear model *F*_1,46_ = 0.91, *p* = 0.35, 95% confidence interval 2.72–5.21 in synthetic water vs. 2.09–4.18 in HA treated water). In comparison, females laid the most eggs in synthetic water without either egg ropes or egg‐scented water (Figure 1a, Overall treatment term *F*_4,105_ = 9.84, *p* < 0.001). All Tukey's tests comparing pairwise treatments were statistically significant (*p* < 0.05; Table 2), other than between control water and egg‐scented water without eggs (first column in Table 2, *p* = 0.17). This shows that females are not responding to chemical cues from egg ropes in isolation. The fewest egg ropes were laid in the treatment with egg‐scented water from five egg ropes (Figure 1a).

**Table 2:** Results of Tukey's tests comparing differences between oviposition in relation to five treatments related to the presence of conspecific eggs (0 or 5) or whether eggs were in egg‐scented water (scented/not scented)

	0 eggs/not scented	0 eggs/scented	1 egg/not scented	5 eggs/not scented	5 eggs/scented
0 eggs/not scented					
0 eggs/scented	<0.01				
1 egg/not scented	<0.01	0.61			
5 eggs/not scented	0.17	0.82	0.09		
5 eggs/scented	<0.01	0.11	0.86	<0.01	

Note

The *p*‐value of all pairwise comparisons is shown from a generalized linear model.

### 3.2 Aim 2: Do females make adaptive oviposition decisions?

In our first experiment, females laid more egg ropes in water from the Chandler wetland than from the three other sites (Figure 1b, Treatment term *F*_3,88_ = 2.81, *p* = 0.04). Only the Chandler and Cheltenham wetlands were significantly different though (Tukey's test *p* = 0.04, all other comparisons *p* > 0.05), and comparable numbers of egg ropes were laid in water from the three other sites. 100% of egg ropes hatched when raised in water from each site.

In our second experiment, we found a significant effect of conductivity in both years, with fewest egg ropes laid (12% of total) in the 10,000 μS/cm water (2016: *F*_3,56_ = 3.20, *p* = 0.03; 2017 *F*_3,88_ = 4.12, *p* < 0.001; Figure 1c). In both years, this result was due to the significant differences between the lowest and highest conductivity concentrations examined (Tukey's test between 200 and 10,000 μS/cm in 2016 *p* = 0.02, between 4,000 and 10,000 μS/cm in 2017 *p* < 0.001; for all other comparisons *p* > 0.05). We found no evidence for a threshold value above which females avoid laying their eggs in particular conductivities. In 2017, similar numbers of ropes were laid in the 6,000, 8,000, and 10,000 μS/cm treatments (note overlapping 95% confidence intervals in Figure 1c).

**Figure 2:**
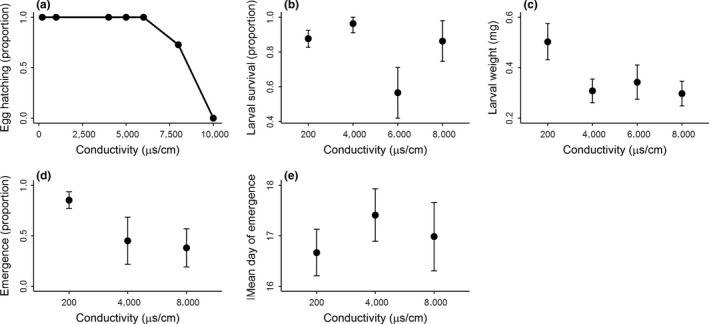
Responses to conductivity in the non‐biting midge (*Chironomus tepperi*): (a) proportion of eggs that hatched in different conductivities, with line showing dose–response curve from log‐logistic model, (b) proportion of larvae that survived at end of 5‐day bioassay, (c) larval weight at end of 5‐day bioassay, (d) the proportion of emerging adults after 18‐day emergence test, and (e) the mean day of emergence in 18‐day emergence test. In b‐e, the mean response (±95% confidence interval) is shown

All egg ropes laid at conductivities below 6,000 μS/cm water hatched successfully, but our modeled dose–response curve showed a rapid decline in the proportion of eggs that hatched at the two higher treatments (Figure 2a). We examined 27 egg ropes laid in 10,000 μs/cm water, and only one of these hatched, with two dead larvae observed after 24 hr. To put this into context, we counted 300–400 larvae moving actively after 48 hr in other treatments. Twenty‐four hours after hatching and being held in this highest treatment, the jelly medium in which egg ropes were contained had completely dissolved.

Larval survival after 5 days was significantly lower at 6,000 μS/cm than 200, 4,000, or 8,000 μS/cm treatments (Treatment term: *F*_3,28_ = 11.56, *p* < 0.01, Tukey's for these comparisons all <0.01, Figure 2b). Larvae held in 200 μS/cm water were significantly heavier than other treatments (Treatment term: *F*_3,28_ = 8.29, *p* < 0.01, Tukey's for these comparisons all <0.01, Figure 2c).

Fewer adults emerged from 4,000 and 8,000 μS/cm treatments (Treatment term: *F*_2,13_ = 13.29, *p* < 0.01, Tukey's for these comparisons all <0.01, Figure 2d). In comparison, the timing of emergence was comparable between treatments when both overall (Treatment term: *F*_2,13_ = 1.79, *p* = 0.21) and sex‐specific timing (Males: Treatment term: *F*_2,11_ = 0.01, *p* = 0.99, Females *F*_2,13_ = 0.12, *p*: 0.89) were considered (Figure 2e, results pooled across both sexes).

### 3.3 Aim 3: Can NHPI exacerbate the effects of ecological traps?

**Figure 3:**
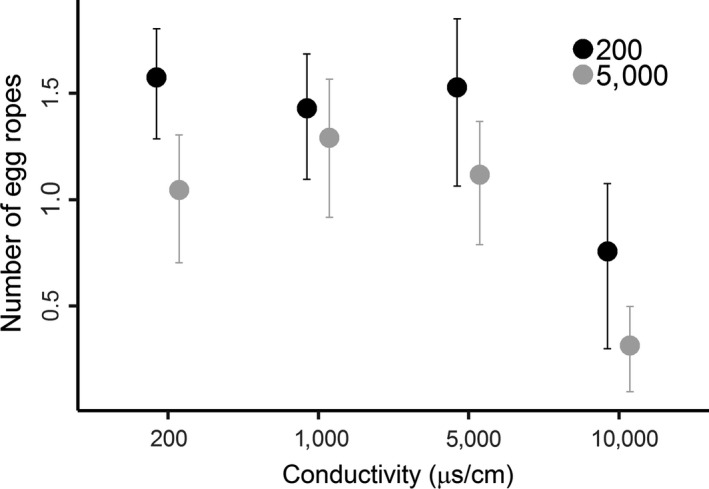
Oviposition behavior of the non‐biting midge *Chironomus tepperi* raised in two natal environments (200 and 5,000 μS/cm conductivity) to conductivity. The response variable is the mean number (±95% confidence interval) of egg ropes laid in each treatment across all replicates, log‐transformed

Oviposition preferences were consistent across the two natal environments; thus, we found no support for natal habitat preference induction (*F*_3,160_ = 0.95, *p* = 0.42). Adults raised in both 200 and 5,000 μS/cm water laid less eggs in the 10,000 μS/cm treatment (Figure 3, Tukey's tests comparing 10,000 μS/cm to other treatments all *p* < 0.001; all other comparisons between treatments *p* > 0.05). However, the natal environment did have an effect on reproductive investment, with females raised in 5,000 μS/cm tending to lay fewer egg ropes irrespective of which treatment these were laid in (95% CI for number of egg ropes laid per cage pooled across treatments: 200 = 8.92–14.50, 5,000 = 5.52–8.27).

## 4 DISCUSSION

### 4.1 Aim 1: Do *C. tepperi* respond to potential oviposition cues?

Theory predicts using multiple habitat selection cues may mean animals are more informed about their environment, and thus less susceptible to ecological traps (Battin, 2004). *C. tepperi* can use several different cues during oviposition, given they respond to conspecifics and conductivity (this study), and other bioextracts (Stevens et al., 2003). Other chironomids also respond to differences in polarized light (Lerner et al., 2008). Collectively, these results highlight that they can use both visual and olfactory sensory modalities to assess potential oviposition sites. This can reduce uncertainty in decision making, as each modality may have limitations when used individually, or provide independent estimates of likely habitat quality (Munoz & Blumstein, 2012).

*Chironomus tepperi* did not respond to HA, which could potentially indicate that females do not respond to cues from decaying organic matter during oviposition. However, there are other explanations. For example, we only tested one concentration of HA, based on studies that have elucidated responses by fish. It is possible that chironomids have different threshold concentrations to which they respond; further tests with a wider range of treatments would allow this possibility to be explored. Alternatively, HA may simulate some aspects of decaying organic matter (e.g., it darkens the water) but not those to which females are responding to (e.g., olfactory cues).

Females may lay fewer egg ropes in the presence of conspecifics to reduce cannibalism, given we observed newly hatched larvae eating each other and the egg rope they hatched from, and help ensure their offspring are the early colonists of newly flooded habitats (Stevens et al., 2003). Some species exhibit density‐dependent responses to conspecifics (Almohamad, Verheggen, Francis, Lognay, & Haubruge, 2010; Raitanen et al., 2014), with attraction at low‐density and avoidance at high. It would be interesting to test whether *C. tepperi* exhibited a similar switch when exposed to a wider range of treatments, as all our treatments could reflect high natural densities.

We found a significant difference in the number of eggs laid in the highest and lowest conductivity treatments in each year but while the highest concentration was 10,000 μS/cm, the lowest was 200 and 4,000 μS/cm in 2016 and 2017, respectively. As the difference in oviposition among treatments was greater in 2016, this suggests that females might use conductivity as a cue but its reliability depends on the range in conductivities they are assessing. There is thus the potential that spatial and temporal variation in the environment can alter the choices animals have in ways that can affect whether selecting a particular habitat is adaptive or not. The concept of spatial contagion provides further support for this idea, where the decision by animals about the suitability of a habitat is influenced by the types of cues they encounter from nearby patches (Resetarits & Binckley, 2009). In the ecological traps literature, studies have tended to document more “severe” than “equal preference” traps (Hale & Swearer, 2016; Robertson et al., 2013) but this raises an interesting question—are “severe” traps more common, or are they reported more often because they are more distinct from nearby patches and thus more easily detected? More tests examining the behavior of animals in relation to more subtle differences between habitats will help assess the relative frequency of “severe” versus “equal preference” traps.

### 4.2 Aim 2: Do females make adaptive oviposition decisions?

The stormwater wetlands we examined do not seem to be ecological traps for *C. tepperi*, at least when egg hatching is used as a fitness endpoint. While females laid more egg ropes in water from the most polluted site, all egg ropes hatched regardless of the water they were raised in. Recent results have also shown that larvae reared in sediments from these sites had high (85%–100%) and comparable survival (K. Jeppe unpublished data). The two more polluted sites here are ecological traps for native frogs (Sievers, Parris, Hale, & Swearer, 2018). The traits and behaviors of animals likely to increase susceptibility to ecological traps have been described, for example, slow rate of evolution, slow generation time, and low capacity for learning (Battin, 2004; Hale, Treml, & Swearer, 2015), and recent work has begun to explore taxonomic variability in the likelihood that closely related animals (e.g., different insect families) are susceptible to ecological traps (Robertson et al., 2017,2018). Less is known about how potential ecological traps affect different taxonomic groups. *C. tepperi* are quite resistant to the effects of metal pollution (Hale et al., 2014), despite larvae inhabiting sediments, but may be more sensitive to other forms of pollution, such as insecticides and herbicides (Phyu, Warne, & Lim, 2005; Stevens, 1992). In comparison, frogs, specifically tadpoles, might be sensitive bio‐indicators of environmental alterations to wetlands (Sievers, Hale, Parris, & Swearer, 2018). More comparisons of the susceptibility of animals with contrasting life histories and behaviors to the same potential ecological traps would help identify which species are most susceptible and why. In particular, this will allow comparisons to be made about whether taxonomically distant species are ecologically trapped by the same or different mechanism.

Females laid fewer egg ropes at higher conductivities, but are they behaving adaptively? We found a dramatic threshold in egg rope hatching success: 100% up to 6,000 μS/cm, ~70% at 8,000 μS/cm, and ~0% at 10,000 μS/cm. In 2017 though, nearly 40% of egg ropes were laid at 8,000 and 10,000 μS/cm, so females are still often choosing locations where their egg ropes fail to hatch (Figure 2c). Females also laid many egg ropes in treatments between 4,000–6,000 μS/cm, where other fitness costs were observed: larvae were smaller, and fewer adults emerged. The unexpected larval survival results we observed (reductions at 6,000 μS/cm but not at 8,000 μS/cm) could be due to a statistical artifact, or a real biological response such as homeostasis or compensatory behavior (Davis & Svendsgaard, 1990); more work is needed to understand the mechanism. The deleterious effects of conductivity on this species have been highlighted in the context of examining its potential confounding effects on detecting sediment pollution (Hale et al., 2014). However, conductivity could be a stressor for *C. tepperi* in its own right, as increased salinization in freshwaters is a global environmental issue (Kefford et al., 2016), and widely distributed species like *C. tepperi* may encounter a range of habitats, including those with salinity issues.

Why do females not avoid locations where offspring fitness is compromised if they have the ability to assess the potential suitability of these locations? Perhaps conductivity is difficult for *C. tepperi* to accurately assess, especially when the choice is between subtle differences (i.e., our 2017 experiments). Female chironomids can select oviposition sites on the basis of polarized light (Lerner et al., 2008), and maybe the polarization of the surface water is the same between all of the conductivities we used. Further work is needed to explore if this is the case.

Laboratory choice experiments are commonly conducted to understand the habitat selection behavior of a diverse range of taxa, but particularly those that inhabit aquatic environments, such as fish and insects. While these methods can provide useful information about the mechanisms underpinning habitat selection (e.g., what cues animals respond to), it is important to acknowledge that test conditions in the laboratory represent a simplification of the natural environment, and thus, it is important to subsequently understand how laboratory results translate into behaviors in the wild. Laboratory trials can inform field trials, particularly in terms of identifying hypotheses to be tested. For instance, based on our results, we would predict that if the types of habitats females encounter in the wild affects their habitat selection decisions, they may be more likely to lay their eggs in high conductivity habitats where they suffer fitness costs if these habitats are not clearly different from nearby sites. Extending our results into field trials (e.g., using microcosms: Pettigrove & Hoffmann, 2005) will be an important next step to explore behavior under more realistic settings. One important consideration, however, is that these methods may be more useful for examining responses for a wider range of species, given that there is no guarantee that a target species will colonize microcosms.

### 4.3 Aim 3: Can NHPI exacerbate the effects of ecological traps?

We found no evidence that oviposition preferences are related to natal experiences, with females from both natal environments avoiding 10,000 μS/cm water. Our results suggest females are ovipositing adaptively in relation to egg survival, given that all egg ropes raised in water with conductivities <6,000 μS/cm hatched. Fewer egg ropes, though, were laid by adults raised in 5,000 μS/cm, so natal experience may instead affect fecundity. Females may breed in environments suitable for some but not all life‐cycle stages, for example, midges generally, but not exclusively, avoid waters with high cadmium where eggs hatch but larvae die (Williams, Green, Pascoe, & Gower, 1987). The consequences of experiencing a poor natal environment could also be passed onto offspring through transgenerational effects (Colombo et al., 2014).

Natal influences on habitat preference vary considerably between taxa (Davis, 2008). To affect habitat selection, either preferences learned in natal environments must be held in memory or the natal environment could have developmental effects on the nervous system, as many insects have complex brain structures that allow both short‐ and long‐term memory of previous experiences (Dion, Monteiro, & Nieberding, 2018). However, we would expect NHPI to be unlikely in holometabolous insects whose nervous system is reorganized during metamorphosis (Davis, 2008). NHPI is also more likely to be adaptive in environments that are temporally stable so past conditions are a good indicator of future conditions. *C. tepperi* inhabit ephemeral habitats, and previous work suggests they do not oviposit into rice fields from which they emerged (Stevens, 1994); thus, NHPI is less likely to be adaptive. For species with complex life cycles where dispersers do not return to natal habitats, a more useful strategy might be to use simple decision rules such as moving along environmental gradients (Ousterhout, Luhring, & Semlitsch, 2014). Having canalized behavioral responses (i.e., making identical choices regardless of natal environment), even if they include laying some eggs in poorer sites, may reduce the likelihood that all progeny are lost than if highly flexible approaches influenced by natal experience are used (Reiskind & Zarrabi, 2013). Further work is needed to explore the proximate mechanism of habitat selection for *C. tepperi*.

## 5 CONCLUSIONS

Some species can alter their behaviors to adapt to HIREC, such as birds adjusting their behaviors in urban areas (Sol, Lapiedra, & Gonzalez‐Lagos, 2013), but many other species fail to adapt. Knowledge about how animals perceive, evaluate, and respond to cues from their environment is a critical component in explaining this variability (Sih, 2013; Sih et al., 2011). *C. tepperi* respond to a range of oviposition cues using at least two sensory modalities and thus should be less likely to select ecological traps. They are also tolerant to some pollutants, such as heavy metals (Hale et al., 2014), which might mean that a range of habitats are suitable. Nonetheless, our results highlight that this species might still be at risk of ecological traps caused by increasing conductivity but the probability of selecting such habitats will be context‐dependent. Our study highlights the four questions that need to be answered to understand whether animals experience ecological traps and why: (a) how does fitness vary between habitats, (b) do animals prefer some habitats, (c) what cues do animals use during habitat selection, and (d) what are the mechanisms underpinning habitat selection behavior? More studies that address these components simultaneously will improve current knowledge of which species respond adaptively or not to environmental change and why.

## CONFLICT OF INTEREST

We have no competing interests.

## AUTHOR CONTRIBUTIONS

All authors contributed to experimental design and writing the paper; RH, VC, and MH conducted the experiments; RH conducted statistical analyses.

## Data Availability

Empirical data has been archived in DataDryad: https://doi.org/10.5061/dryad.d20221b
